# Family matters in Canada: understanding and addressing family homelessness in Ontario

**DOI:** 10.1186/s12889-022-13028-9

**Published:** 2022-03-29

**Authors:** Cheryl Forchuk, Gordon Russell, Jan Richardson, Chantele Perreault, Heba Hassan, Bryanna Lucyk, Sebastian Gyamfi

**Affiliations:** 1 Western University, Nursing, London, ON Canada; 2 Lawson Health Research Institute, London, ON Canada; 3 Parkwood Research Institute, London, ON Canada; 4 STN B, Beryl and Richard Ivey Research Chair in Aging, Mental Health, Rehabilitation and Recovery, Mental Health Nursing Research Alliance, Lawson Health Research Institute, Parkwood Institute Mental Health Care Building, 550 Wellington Road, Suite B3-110, P.O. Box 5777, London, N6A 4V2 Canada; 5 Mission Services of London, London, ON Canada; 6 London, Canada; 7 Rotholme Women’s & Family Shelter-Mission Services, London, ON Canada

**Keywords:** Homelessness, Family, Shelter, Strategies, Homeless, Canada

## Abstract

**Background:**

Homelessness is becoming an international public health issue in most developed countries, including Canada. Homelessness is regarded as both political and socioeconomic problems warranting broad and consistent result-oriented approaches.

**Methods:**

This paper represents the qualitative findings of a project that explored risk factors associated with family homelessness and strategies that could mitigate and prevent homelessness among families using a focused ethnographic study guided by the principles of participatory action research (PAR). The sample includes 36 family members residing at a family shelter who participated in focus groups over two years (between April 2016 and December 2017). Most of the participants were single-parent women.

**Results:**

The analysis yielded five major themes including, life challenges, lack of understanding of the system, existing power differentials, escaping from hardship, and a theme of proposed solutions for reducing family homelessness in the community.

**Conclusion:**

The findings illustrated the complex nature of family homelessness in Ontario; that the interaction of multiple systems can put families at risk of homelessness. Findings from this study underscore the need for urgent housing protocols aimed at educating homeless families on how to navigate and understand the system, enhance their conflict resolution skills, and develop strategies beyond relocation to help them to cope with difficulties with housing.

## Background

Family homelessness is a global problem that has warranted unique preventive strategies in several countries, including the USA, United Kingdom, Denmark, Finland, Ireland, Netherlands, Norway, Portugal, Sweden, and Australia [1, 2]. The Canadian Observatory on Homelessness (COH) defines homelessness as the situation of an individual or family without stable, permanent, appropriate housing, or the immediate prospect means and ability to acquire it [3]. The COH identifies four types of homelessness: 1) the unsheltered; persons who are absolutely homeless and living on the streets or in places not intended for permanent or private human habitation, 2) the emergency sheltered; comprising persons in overnight shelters and those in crisis such as from family violence, or natural disasters such as fires or floods, other people experience temporary accommodation; 3) staying in interim or transitional housing, or living temporarily with others, or in some other type of housing (e.g. motels, hospital, or prison) without a permanent housing plan, the ‘at risk of homelessness’; and 4) people who are not homeless but have a poor economic or housing situation that is below public health and safety standards [3]. Family homelessness is becoming a public health issue in most developed countries, including Canada. Family homelessness is on the rise due to systemic or societal barriers, such as lack of affordable and appropriate housing, individual/household finances, as well as mental, cognitive, behavioral, or physical challenges, and racism and discrimination [3, 4].

Among the homeless are Canadian families with dependent children, a very vulnerable population that is hardly mentioned in the homelessness literature. Families with children are the fastest-growing homeless subpopulation in Canada [5, 6], leading to high patronage of emergency shelters. Families with dependent children are three times more likely to stay longer in emergency shelters compared to the rest of the homeless population [7, 8]. A recent report by Employment and Social Development Canada (ESDC) demonstrates that family shelters account for 11% of all shelter beds in Canada [9]. The COH [3] reported that most people (including families) experience negative, unpleasant, stressful, and distressing situations concerning their homelessness. The worsening situation of homelessness family appears to be due to systemic failure to serve and support families in difficult transitions from child welfare, discharge planning, (inadequate discharge planning from hospitals, correction centers, mental health, and addictions facilities), and lack of support for immigrants and refugees. Furthermore, structural challenges of inadequate income, lack of affordable housing, and family violence contribute to family homelessness [5].

It is also reported that homelessness families suffer from challenging issues of health and wellbeing [10]. This happens perhaps due to long term poverty, which brings them (mostly mothers and children) into contact with environmental risks such as violence, abuse, and exploitation [11–14]. Mothers who experience chronic homelessness are more likely to suffer from substance abuse, depression, suicide or suicide attempts, while the children may experience poor academic performance, interrupted growth and development, behavioral problems, hunger and social withdrawal [15, 16]. Homeless women may also be exploited financially by friends and other acquaintances they meet or stay with [17], which may lead to physical, emotional, psychological, and financial difficulties. According to Pottie and colleagues [18], “Case-management interventions, with access to psychiatric support, are recommended as an initial step to support primary care and to address existing mental health, substance use and other morbidities” (p. E240) among the homeless.

Studies that examine family homelessness are vital to public health because, as common knowledge, housing issues constitute a part of the physiological paradigm of fundamental human needs towards healthy growth, and thus, form a key part of the social determinants of health. We believe, finding solutions to family homelessness will improve public health and wellbeing of the general population. Generally, there is a paucity of published literature that have studied the experiences of homeless families in Canada. Even though few recent studies have explored family homelessness experiences in Canada [19, 20] with the shifting epidemiology of homelessness towards families with dependent children [15] it has become necessary to intensify research among these unique population. Family homelessness is on the increase with all of these negative effects, but yet continues to receive less attention compared to the general homeless population [21]. Even though researchers have highlighted the need for preventative strategies and enhanced social support for families in need [5, 21–23] the strategies associated with the successful prevention of family homelessness remain understudied. It will, therefore, be novel to look at the needs of families alongside associated risk factors and the strategies that could help maintain housing stability among Canadian families with children. This study, being the first to be undertaken in London Ontario, was therefore designed to explore the needs of homeless families, to identify risk factors associated with family homelessness, and strategies that could assist in mitigating and preventing homelessness among families. This study sought to address two questions: (1) what are the factors and situations that families at risk of homelessness perceive to put families at risk of homelessness? (2) What are the strategies and solutions recommended for reducing family homelessness?

## Methods

### Design

The current study explored the needs of homeless families to identify risk factors associated with family homelessness, and strategies that could assist in mitigating and preventing homelessness among families. This paper reports qualitative data from a focused ethnographic study guided by the principles of participatory action research (PAR). A participatory approach requires researchers to conduct research “with” rather than “on” the people of interest, allowing the voice of those affected by the systems under study to be heard [24, 25]. Researchers worked with participants to collaboratively investigate issues ranging from problem identification to solution implementation [26].

A focused ethnography approach was used for data collection in this study. Focused ethnography allows the researchers to focus on a particular social phenomenon within a shorter period of time than traditional ethnographies [27–30]. This approach is usually conducted with a specific problem in a particular context and is performed with a group of people who share the same experience (in this case homelessness) without being identified with the same culture [31]. This helped researchers to focus on some emic perspectives; the distinct and shared experiences by families concerning homelessness in Canada [27, 32]. Further, this approach is suitable for studies involving persons who are vulnerable [30, 33]. We undertook focused ethnography by focusing on the specific issues surrounding homelessness voiced by the participating homeless families.

### Sample

Over two years, two focus groups were conducted each year with different groups of family members who were accessing an emergency family homeless shelter in Ontario, Canada (a total of 36 families who did not call the shelter first and were not offered the prevention program) but ended up residing at the family shelter took part in the study between April 2016 and December 2017). Most of the participants were single-parent women between the ages of 18 to 59 years.

### Data collection

The study was approved by the Western University Research Ethics Board (REB). Prior to participating in the study, the researchers recruited and obtained written informed consent from each participant.

All methods relating to the study were carried out in accordance with the REB guidelines and regulations.

The family shelter prevention program employed a Housing Crisis Worker to assist families at imminent risk of homelessness. Posters announcing the research study were distributed in the shelter for participant recruitment. Family members contacted the researchers to be included in the focus group. Before the commencement of data collection, the researchers reiterated the objectives of the study, after which they obtained informed consent from each participant before starting the focus groups. Qualitative data was collected through focus groups with people who did not access the prevention program and ended up in the shelter. Focus groups took an average of 90 min each. Data were audio-recorded and then transcribed verbatim by two members of the research team. Note-takers gathered information about group dynamics, context, and non-verbal information, which were integrated into the transcribed data to augment research findings.

### Data analysis procedure

A qualitative analysis inspired by Leininger [34] was applied in examining the data from the focus groups conducted with families at a Family Shelter. Leininger’s ethnonursing data analysis technique involved four steps; included (a) collecting data through interviews, observations, recordings, transcriptions, participatory activities, and field notes. (b) recording and classifying the collected data. (c) identifying, categorizing, and reviewing the data, and (d) interpreting and synthesizing the findings into major themes, with confirmation from the study participants. Two members of the research team initially identified descriptors. This facilitated the evaluation of similarities and differences existing across focus group transcripts [34]. From the descriptors, recurrent themes were identified, and distinct categories formulated. Other members of the research team verified the completeness of data analysis by cross-checking transcripts against formulated categories and resultant themes. Finally, all co-researchers evaluated the preliminary results at a meeting, after which all comments were gathered and incorporated into the study results. After incorporating all comments from the research team, true to the PAR approach, the principal investigator presented the findings that emerged to the participants at a public event to share and establish the validity of the study findings. After perusing the findings, the participants made comments about the quotes. This helped to establish trustworthiness and credibility of the study findings. Through member checking, the participants of the study had more control over the data collected, and thus were able to authenticate the findings by evaluating and making suggestions. After assessing the themes for internal homogeneity and external heterogeneity i.e., data coherence within themes and distinctions between themes [35], we categorized the identified subthemes into five overarching themes.

## Findings

**Fig. 1:**
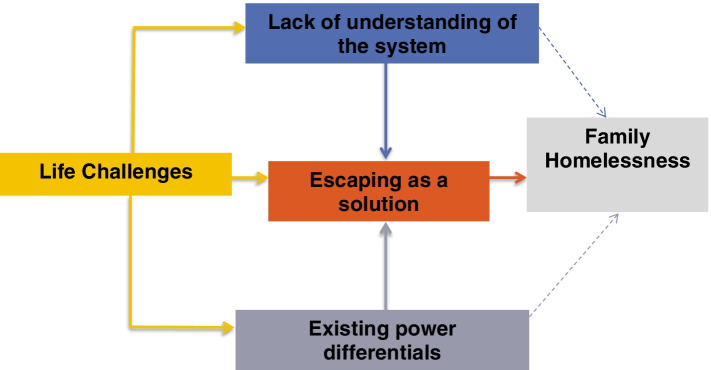
Pathways to family homelessness

This paper reports the qualitative findings from homeless families residing in a shelter at the time of data collection who participated in focus groups. The qualitative findings revealed that there are certain pathways most families pass through, leading to homelessness as well as strategies for dealing with family homelessness. These pathways involve four major themes, which include: 1) life challenges; 2) lack of understanding of the system; 3) existing power differentials, and 4) escaping from hardship. An illustrative diagram of the pathways to family homelessness is presented in Fig. 1 below:

### Theme I- life challenges

Participants’ expressions during focus group discussions were primarily predisposing psycho-socioeconomic difficulties that could be exacerbated by having limited knowledge of systems, structures, and regulations, or limited conflict resolution skills. Families may not know how to navigate the systems, structures, and rules around them, or may perceive themselves as having limited options to change their circumstances. Participants identified the following life challenges:

#### Mental health issues

Some participants disclosed that they had issues with mental illness. According to them, their illness caused them to either become unemployed or spend a lot of money daily, leading them to becoming bankrupt.

A participant shared this viewpoint:

With depression, for instance, people that are depressed tend to go out and spend money on say like fast food because it’s too much to cook or to buy stuff for their kids because they are outside… bad credit. I had great credit, I ended up having a nervous breakdown and couldn’t work for a while, and all bills got backed up and never been able to catch up. And so that’s all because of what happened as a child with abuse and sexual abuse.

#### Lack of social support

Some participants were not very happy with the lack of support in the social domain. They expressed that in the absence of close relatives, no one from the community was ready to assist them in meeting their rent requirements. Some experienced severe distress as a result. During focus groups, participants intimated “A lot of people can’t get guarantors if they don’t have family. When they come from no one and have nothing, how are you supposed to get anything at all?” “They told us at the housing building – that had everything that we need, and then they turned around and told us that we never gave them everything needed and that’s why we were kicked out”.

#### Low income

Financial problems seem to lead a lot of participants into homelessness. This was expressed by most participants. For instance, the following individual said:

So like for me it’s recognizing that yeah your bills need to be paid and I don’t always do that because we don’t necessarily have all the money for the stuff that they want, which just furthers the poverty and homelessness.

#### Inadequate budgeting

Some participants felt their actions and inactions might have contributed to homelessness. They believed inadequate planning of their finances underwrote their current state of homelessness. For instance, this participant hinted “Well yah, it [inadequate budgeting] would affect your credit for certain, because if you don’t manage finances because you are not in the proper education to know how to do that then that can contribute”.

#### Unemployment and cycle of poverty

Joblessness was widely linked to clients’ cycle of poverty. They believed that having a low level of education affected employment opportunities available for them, thus, culminating in homelessness. A participant disclosed:

Well if you don’t have at least one year of any type of postsecondary now it is very hard to get a good paying job. You are in jobs that are geared towards teenagers, they are minimum wage, low paying and you can never get further ahead.

Another participant said, "So people that have grown up in poverty tend to raise their kids in poverty and the cycle continues and there has to be a way to [dealing with it]”.

### Theme II- lack of understanding of the system

The study participants explained that their lack of understanding of certain structural factors and situations put them at risk of homelessness: These factors included lack of awareness of the rental system and issues with social assistance.

#### Lack of awareness of rental system

Some participants felt that a lack of knowledge about rules and processes governing rent led to their homelessness. While some participants blamed the shelter staff for not informing them about the existence of rent banks (Canadian homelessness prevention service that provides interest-free loans to at risk low-income households to pay for specific things for housing stability such as rent or utilities in arrears). In addition, most participants complained of unreasonable rent requirements from landlords. For instance, a participant intimated that a landlord required her to provide up to date monthly child social benefit (baby bonus) slips or receipts that they have been getting from the government. They believe the actions of these landlords made it difficult for them to rent a place due to the high cost and associated challenging demands. A female participant disclosed:

I’ve been at shelter for almost a month, and I’ve viewed maybe 25 apartments, and I finally found one. They told me, ‘you need to bring in your baby bonus slips to know how much you’re getting’. I didn’t do that and I didn’t know that before, I mean, rent’s $850 … my cheque’s $950, so the landlord’s looking at ‘So you’re going to have $100 to live off at a month’, not even thinking that I need to hand in all my other income in order to know that I’m getting more income, but just that they don’t tell you, our worker didn’t even let us know about the rent bank. I didn’t know it existed.”

The participants shared similar experiences during focus group discussions. One of the participants said, "This landlord wanted me to give first and last month's rent before he even approved my application".

#### Issues with social assistance

Some participants complained that they were described as ‘being ineligible for Ontario Works’ (general welfare) due to having worked the previous month despite being unemployed at the time of seeking for support. These participants expressed their frustration in the following ways:

I wasn’t on assistance for years and years and years I worked. Um, I was….part time. My husband at the time, lost his job. We went for help and they told us that because we had made money… that we were not eligible. I had no money for rent, no money for bills, I’m on the street. I was literally homeless for two and a half months before I found a job …and before I could even go to welfare. And I said to them ‘This program is not – It was supposed to be set up for situations like this. Yeah, I made money last month. I paid my bills last month. I don’t have any money to pay my next bills. So because I worked, now I’m being penalized? And now I’m on the street?

Some participants disclosed that the social assistance process is cumbersome. This, according to the participants contributed to their homelessness. One female participant shared:

Unless you have an application [Ontario works] filled out you can’t get that paper signed [lease papers], and until you get an application you can’t get the stability bank, so it all runs in together and you’re already done what you need to do, but, like, the government and all their stuff is behind so you just feel defeated, like you can’t do anything.

### Theme III- existing power differentials

Findings from the focus groups revealed that participants were powerless when it came to negotiating with homeowners. Some participants lacked the skills necessary for dealing with dissension. This led to interpersonal obstacles that prevented them from obtaining or retaining housing, leading to homelessness. Some of the factors that contributed include:

#### Gender and sex-related conflicts

Focus groups revealed that gender preferences and sexual orientation issues worked against some participants in their search for housing. Participants reported experiences of alienation with their own families as well as skirmishes with their landlords that led to their homelessness. A participant disclosed:

If someone was gay and their parents don’t quite like it, they could, ‘get out of my house’ I know someone personally who’s been kicked out of their parents’ house because they got with someone of the same gender and their parents were just not having it.

Another participant added:

I know a transgender female, who’s a mother and she has all kinds of problems because she’s still in the process of changing genders and when her landlord found out…because when she first moved in there she was male, and through the process of becoming female, she adopted the baby and wanted to upgrade to a two-bedroom from the one-bedroom and landlord lost it and refused. It was a long court battle and she won but she still had to find a new place.

#### Race-related issues

Some participants disclosed that even though they were citizens, they faced racial discrimination from landlords during their housing search. This perceived discrimination from landlords contributed to their housing difficulty. A participant disclosed:

“The landlord don’t want to rent to anybody colored”. Another participant added their voice to support the apparent racial denigration from some landlords; “The landlord was making like a slur of something of that race, or whatever”. This participant also disclosed how a landlord behaved towards her and her partner in their housing search.

#### Unclarified landlord-tenant responsibilities

Some tenants and their landlords were at loggerheads with each other. This disharmony either led to tenants moving out or being evicted by landlords**.** For instance, this parent intimated “Yeah! If my fridge breaks down and I call my landlord, is he gonna turn around and say, ‘you’re fixing it yourself’ or ‘I’ll fix it? But I’m gonna dock rent’.

### IV- Escaping as a Solution for Hardship

When significant problems or aversive events occurred, families described ‘Escaping as a solution for hardship’. Relocation was described as the primary method used by many families to cope with the following hardships:

#### Family and relationship issues

Participants explained that strained relationships with their families led to their homelessness. One participant explained:

So is that how I’m gonna start thinking soon, I should’ve waited longer? I feel like I moved out when I was ready, like my parents kept kicking me out. They prepared me for it when they officially finally said, ‘don’t come home’ and you know my parents did it too many times, so when they said the last time, ‘you need to find somewhere to go and stay’, I said fine, I‘m finding somewhere to go and stay and I’m not coming back.

Another participant added; “Like even when I lived with my parents, my mom had mental health issues, and she has the same ones as I do. We butt heads all the time, so that’s why we’ve constantly been kicked out.”

#### Difficulty getting housing

The high cost of rent and bad credit made it difficult for some families to acquire accommodation. Some participants became frustrated as a result.

For instance, this participant disclosed; “Rent is expensive. Welfare don’t give you much. Yeah, hydro and electricity are ridiculous.” This next participant expressed how bad credit history led to their homelessness; “Yeah! A lot of places they, they want that up front now, which is good like on their behalf and I understand, but at the same time if you don’t have established credit yet something they’re not looking into.” Another focus group participant added; “Your credit definitely, like for me that was mine.”

#### Perceived adverse housing conditions

Some families were concerned about perceived unfavorable neighborhood or housing conditions. While some participants raised issues about insecurity in the neighborhood, others complained about irresponsible landlords. Most participants complained about how landlords were shedding their responsibilities. This made them uncomfortable and led them to move out even in the absence of a potentially safe place of abode. The only option for them was to live on the street or go to a shelter.

A participant shared:

I think dwelling is one of the factors, like the places that we have come from had mold and bed bugs problems that have gone untreated and just landlords that just don’t care. So that was more reason for us not to want to try to and work with that landlord or anything like that.

Another participant revealed:

Yeah, they weren't treating on a consistent basis to address the problem. Yeah, we came home to [See] blood all over the hallway. And that contributes…to being homeless because you don't want to pay rent somewhere where you don't feel safe and you don't feel like they are addressing problems that you say to.

### Theme V- Participants’ proposed Solutions for Reducing Homelessness

A fifth theme, separate from the pathways to homelessness emerged in response to the question of ‘which strategies and solutions are recommended for reducing homelessness?’ This theme was ‘participants’ proposed solutions for reducing homelessness in the community’. Interactions between researchers and participants uncovered seven strategies that could assist in lessening family homelessness. These sub-themes include; certification/training for renters, affordable and safe housing, re-examining social assistance’s processes, linking people to supportive services, transitioning off of social assistance to employment. The rest are greater understanding and compassion from social service workers and ensuring access to resources.

#### Certification/training for renters

Some participants suggested that both renters and landlords be educated including persons who are currently homeless on budgeting, finances, and tenancy-related issues. According to the participants, being knowledgeable in tenancy issues will help renters make informed housing choices whiles landlords are likely to demonstrate restraint and empathy towards tenants. During focus groups, a participant expressed this view;

In Ontario and Canada renting to anybody should have to have a license to do so. They should have to go through some sort of a test, some training program sort of…, so that [you] can’t just go ahead and buy a house and rent it out to whomever [you] want. You know what I mean?

Another participant intimated; “They should have compassion training [referring to the landlords].” This next participant suggested education for tenants to forestall future homelessness. “Try to educate them [referring to homeless people) through finances or budgeting or … try to educate them before it comes to the point of homelessness”.

#### Affordable and safe housing

Even though the participants admitted the existence of affordable housing programs, they bemoaned the unsafe living conditions coupled with negative public perceptions towards the occupants of these houses. Some participants suggested that the housing should be both affordable and safe to provide them the comfort they need to be able to take care of their children. One of the participants expressed this opinion:

Affordable housing is great but if maintenance and everything else is not caught up people are not going to care to live there and then the cycle starts all over again. It needs to look presentable not just for your tenants but society as a whole. Because society as a whole looks at [us; the homeless] ‘oh they are scum, they are nothing but drug addicts’, and others in affordable housing get the same. Like, a lot of people… it’s not just us, I know a lot of people don’t want to look at affordable housing because there’s such stigma there as well as when geared to income that their ‘bad neighborhoods’, you hear the word, and everyone’s like I don’t trust my vehicle there. My aunt came to visit there once, she did not trust [us], she was afraid that her hubcaps were going to get stolen.

Another participant also reiterated:

Habitat for humanity …it’s build people houses that have been living in inadequate or improper housing, but you have to have a set income and a certain debt load in order to qualify, and that’s great but it doesn’t help the people that are below that amount, that need that actual stable housing, it helps basically the middle class… They are already stable you need to help the below middle class, and most of the tax breaks are for the middle class and up.

#### Re-examining social assistance’s processes

During focus group discussions participants called for the re-assessment of existing social assistance procedures. They advocated that officials should attach a human-face on the processes involved in acquiring assistance with housing. A participant disclosed:

Affordable housing is great in theory but the problem is you base RGI (rent geared to income) on the gross, the person does not get the gross, the person gets the net, so therefore if you base it on the gross…..say one month you work, and you have statutory holiday pay, they base next month’s rent on the previous month, what if that month does not have a statutory holiday on it? So you are paying a higher amount of rent based on the previous month when you don’t have that income.

#### Linking people to supportive services

Most participants also recommended that there should be an effective collaboration across services to enhance ease of flow of assistance between multiple programs. This way, housing interventions could be sustained. One participant recommended; “Maybe the social assistance workers…need more responsibility… they should be more helpful… they should be in contact with the Rent Bank”. This participant also added this view; “Well, even now, you need…. last month’s rent to get an apartment, so maybe there can be like a voucher? Why is there a rent bank? Why can’t it just go through social assistance?”.

#### Transitioning off social assistance to employment

During focus groups, participants expressed that individuals who have been recently employed be given ample time to work until they are in a stable situation before they are taken off the social assistance system. To them, this will act as an incentive for anybody on assistance to go and get a job. A participant expressed this sentiment:

To be perfectly honest the biggest thing that needs to be changed is that people who are capable to work can work until they are in a stable situation where then they can be taken off welfare. But what I can’t see is how I’m supposed to go out and get a job, right? When I’m looking for housing and have them telling me as soon as I get that job, ‘Well, all right, you’re now going to be making $25,000 a year, we don’t care what you’ve made to this point, you’re off welfare.’

Another participant added:

Yeah, transitions to work! I should be able to make any amount of money that I can make for the first month or so anyway, and then have them reassess my situation after a couple of months and say, ‘Ok, you’re stable, your bills are paid, now you no longer need this [support].

#### Greater understanding and compassion

While most participants called for empathy from social service workers, they wondered whether social workers understood their situation. This participant emotionally suggested: “Maybe, like, not so much thinking about money…and like, maybe, having a heart. I think people are in difficult situations… they (social service workers) should not make it so hard, not being ‘so rude’.” This participant also poured out emotions this way; “Yeah, have someone sort of pretend to go through the process and see how that feels like”.

#### Ensuring access to resources

During focus groups, most participants suggested that at the shelter, authorities could make resources available to families such as helping them attain jobs, childcare, or providing needs assessments so that they can be prepared to retain their housing when they leave the shelter. This following participant suggested:

I think when you come into shelter here – I mean, not when you first come in, ‘cause everyone’s in a different mindset – but there should be like a full-out assessment. Like, ‘Do you need help with this? Do you need help with this?’ Like, do you need, like, links to jobs? Do you need a family doctor? Do you need an addictions counselor?

Another participant also added:

They should help people get jobs, you know, help people – Not penalizing for working enough, not penalizing them when they’re worked and they need help. When you’ve paid your taxes into the government and then when you need help they’re telling you ‘No’! You can’t get help until you’re absolutely at rock bottom.’ You know?

## Discussion

This paper reports on the findings of a qualitative study that explored risk factors associated with family homelessness, and the strategies that could help mitigate and prevent homelessness among these families. After completing thematic analysis on the collected data, five (5) major themes were identified: these include life challenges, lack of understanding of the system, existing power differentials, escaping from hardship and proposed solutions for reducing homelessness in the community.

Homelessness is an extreme form of poverty that is shaped by prolonged exposure to environmental risks [10]. Most Canadians including families with children are at risk of becoming homeless due to poverty, personal crises, and lack of affordable housing [5]. Even though Canada has over the years improved on shelters and transitional housing programs, this has not worked in favor of families with children. Instead, these shelters have been found to hinder family processes concerning childcare and upbringing [2] Instead of investing in strategies for managing homelessness, focusing on preventative strategies is paramount [36, 37]. For instance, the participants of the current study indicated inefficient social assistance regulations and processes, undesirable/unsafe housing conditions, difficulty with rent demands, and lack of awareness about services as situations that can put families at risk of homelessness. The COH [3] noted that homelessness was on the rise due to systemic or societal barriers, including, discrimination, lack of affordable and appropriate housing, as well as the individual’s household’s financial standing. Some recent studies [5], and [14] have also asserted that family homelessness occurs due to structural challenges, including inadequate income, and lack of affordable housing.

Affordable housing may not be the only solution to homelessness, it is admissible however that homelessness cannot be solved without adequate and long-term supply of affordable housing; a plan that [5] have supported since 2013. Again, the study revealed psycho-socio-economic factors such as mental health and addiction issues, low income, job loss, poverty, and lack of social support, as aspects that contribute to family homelessness. These findings are in line with assertions by COH [3] as well as [10], that family homelessness may be exacerbated by the psychiatric illness of a parent (usually the head of the family) leading to job loss, depletion of finances and the inability to pay for rent resulting in eviction. Again, inadequate income and lack of affordable housing have all been confirmed by the current study as pivotal in bringing about family homelessness [5, 10]. Family homelessness is a sign of societal failure in making sure that effective systems of funding and support are available for all people in need [3].

Over the years, researchers have focused on strategies for managing homelessness instead of approaches for ending it [36, 37]. Study participants suggested some strategies that could assist in lessening family homelessness while accelerating access to stable housing for families with children. Participants called for training and certification of renters on tenancy-related issues to enhance their knowledge towards restraint and empathy. The study participants proposed the provision of resources such as affordable and safe housing and a variety of housing support programs to hasten access to permanent housing for families. These findings are in line with other study results in recent times such as [5, 21–23] who have also called for enhanced social support for the most vulnerable. Therefore, there is a need to bring all services together in a unified approach perspective that includes upstream and downstream interventions.

### Implications

In the short-term, we expect the pilot program to help families avoid homelessness. By connecting families with these supports, we expect in the medium-term, families will have more stable housing which will improve (or at least maintain) their quality of life, community integration, health, social, and justice service utilization, and child health indicators (mental health, school attendance). In the long-term, we believe the PHAF intervention to be implemented in the municipality and that this will reduce homelessness among families in London, saving money through a reduction in shelter costs. The risk factors and the strategies identified by the homeless families could assist in formulating preventative programming into the City of London’s municipal sectors and also facilitate the City’s development of neighborhood hubs.

### Limitations

Although the step-by-step analysis techniques of Leininger [34] applied in this study enhanced the credibility and transferability of the study findings to other similar settings, the use of qualitative participatory design involving only sheltered homeless families may not represent the entire voice of homeless families who could be staying with other relatives, friends, or on the streets. Therefore, we do not intend to generalize our results beyond similar participants in Ontario. We however intend to undertake future studies in this area involving homeless family country wide.

## Conclusion

This study explored the needs of families at risk of homelessness in a shelter intending to find strategies and solutions for reducing homelessness among families with children.

Major themes from focus groups conducted with a section of homeless families indicate that the path to family homelessness is characterized by common risk factors and situations. The study identified life challenges, lack of understanding of the system, difficulty with conflict resolution and escaping from hardship as major contributing factors to family homelessness. In the end, participants proposed strategies that could assist in lessening family homelessness. These strategies include: Requiring certification/training for renters, providing affordable and safe housing, re-examining social assistance’s processes, linking people to supportive services, transitioning from social assistance to employment, greater understanding and compassion from social service workers, and ensuring access to resources. Findings from this study emphasize the need for further studies involving multiple sites (shelters) provincially to ascertain strategies that can be geared towards enhancing protocols for averting family homelessness in Ontario.

## Data Availability

Datasets generated and/or analyzed during the current study are not publicly available due to participant privacy. Data is however available from the corresponding author on reasonable request.

## Abbreviations

COH: Canadian Observatory on Homelessness
ESDC: Employment and Social Development Canada
PAR: Participatory action research
PHAF: Prevention of Homelessness Among Families
